# A greater lymph node yield is required during pathological examination in microsatellite instability-high gastric cancer

**DOI:** 10.1186/s12885-021-08044-8

**Published:** 2021-03-25

**Authors:** Zhenghao Cai, Haiqin Song, Abe Fingerhut, Jing Sun, Junjun Ma, Luyang Zhang, Shuchun Li, Chaoran Yu, Minhua Zheng, Lu Zang

**Affiliations:** 1 Department of General Surgery, Ruijin Hospital, Shanghai Jiao Tong University School of Medicine, No. 197 Ruijin Er Road, Shanghai, 200025 P. R. China; 2 Shanghai Minimally Invasive Surgery Center, Shanghai, P. R. China; 3 Section for Surgical Research, Department of Surgery, Medical University of Graz, Auenbruggerplatz 29, 8036 Graz, Austria

**Keywords:** Microsatellite instability, Gastric cancer, Lymph node count

## Abstract

**Background:**

The impact of microsatellite status on lymph node (LN) yield during lymphadenectomy and pathological examination has never been assessed in gastric cancer (GC). In this study, we aimed to appraise the association between microsatellite instability-high (MSI-H) and LN yield after curative gastrectomy.

**Methods:**

We retrospectively analyzed 1757 patients with GC undergoing curative gastrectomy and divided them into two groups: MSI-H (*n* = 185(10.5%)) and microsatellite stability (MSS) (*n* = 1572(89.5%)), using a five-Bethesda-marker (NR-24, BAT-25, BAT-26, CAT-25, MONO-27) panel. The median LN count and the percentage of specimens with a minimum of 16 LNs (adequate LN ratio) were compared between the two groups. The log odds (LODDS) of positive LN count (PLNC) to negative LN count (NLNC) and the target LN examined threshold (TLNT_(x%)_) were calculated in both groups.

**Results:**

Statistically significant differences were found in the median LN count between MSI-H and MSS groups for the complete cohort (30 vs. 28, *p* = 0.031), for patients undergoing distal gastrectomy (DG) (30 vs. 27, *p* = 0.002), for stage II patients undergoing DG (34 vs. 28, *p* = 0.005), and for LN-negative patients undergoing DG (28 vs. 24, *p* = 0.002). MSI-H was an independent factor for higher total LN count in patients undergoing DG (*p* = 0.011), but it was not statistically correlated to the adequate LN ratio. Statistically significant differences in PLNC, NLNC and LODDS were found between MSI-H GC and MSS GC (all *p* < 0.001). The TLNT_(90%)_ for MSI-H and MSS groups were 31 and 25, respectively. TLNT_(X%)_ of MSI-H GC was always higher than that of MSS GC regardless of the given value of X%.

**Conclusions:**

MSI-H was associated with higher LN yield in patients undergoing gastrectomy for GC. Although MSI-H did not affect the adequacy of LN harvest, we speculate that a greater lymph node yield is required during pathological examination in MSI-H GC.

## Background

Gastric cancer (GC) is the third leading cause of cancer-related death worldwide [1]. While systemic and multimodal treatment have been widely applied in GC, radical surgical resection remains the cornerstone of curative treatment [2]. Adequate lymph node (LN) yield, an essential quality measure for radical resection, requires a minimum of 16 LNs for precise staging [2–4].

Several publications have found that the munber of LN yield in colorectal cancer (CRC) depended not only on the extent of lymphadenectomy performed by surgeons but also on some tumoral characteristics, especially the microsatellite status [5–8]. In GC, microsatellite instability-high (MSI-H) tumors have also been identified as one of the main subtypes [9]. Although many clinical and pathological characteristics of MSI-H phenotype in GC seem similar to those in CRC, we were unable to find any studies that mentioned the impact of MSI-H phenotype on LN yield. In this study, we aimed to examine whether there was any correlation between MSI-H and adequate LN yield after gastrectomy.

## Methods

### Patients

We conducted retrospective analysis on the records of 1948 patients who underwent gastrectomy for GC at Ruijin Hospital, Shanghai, People’s Republic of China, between 2017 and 2019. All data were collected from our prospective database and the study was approved by Ruijin hospital ethics committee. We included patients with adenocarcinoma, mucinous adenocarcinoma, and signet ring cell carcinoma, excluding those with synchronous double cancers or metastatic GC, those treated with non-curative (R_2_) resection, and those with unstructured pathological evaluation reports.

### Microsatellite status

Four mismatch repair (MMR) proteins, MLH1, MSH2, PMS2, and MSH6, were tested by immunohistochemical (IHC) analysis. Tumors with defective MMR protein expression were considered as MMR deficiency (dMMR). A polymerase chain reaction (PCR) method with a five-Bethesda-marker (NR-24, BAT-25, BAT-26, CAT-25, MONO-27) panel was used for dMMR specimens. Tumors with instability at two or more of the five markers were classified as MSI-H whereas other tumors were classified as microsatellite stability (MSS) in our study [10]. The microsatellite status of positive LNs was not tested.

### LN retrieval and evaluation

After fixation of the en-bloc specimen in 10% formalin for 48 h, gross dissection of LNs was performed by palpation and thin slice inspection. The specimen was routinely re-examined when fewer than 16 LNs were dissected after primary gross examination. Pathological results were reported in line with the AJCC Cancer Staging Guidelines 8th edition [4]. The pathologists involved were unaware of the future inclusion of specimens in this study at the time of assessment.

We defined the adequate LN ratio as the percentage of specimens with a minimum of 16 LNs during pathological examination. We defined the log odds of positive LNs to negative LNs (LODDS) as: LODDS = log [(PLNC+ 0.1)/(NLNC+ 0.1)], in which PLNC/NLNC means positive/negative lymph node count, respectively. We defined a target LN examined threshold (TLNT_(x%)_) as the minimum number of LNs yielded to detect a given percentage (X%) of cases with positive LN (LN_+_). We can calculate the TLNT_(x%)_ from the distribution of LODDS, using this formula: LODDS_(1-X%)_ = log [(1 + 0.1)/(TLNT_(X%)_-1 + 0.1)]. → TLNT_(X%)_ = 1.1/*e*^LODDS(1-X%)^ + 0.9 .

### Statistical analysis

EpiData 3.1 (a free software available at www.epidata.dk) was utilized for data collection. Statistical Package for the Social Sciences (SPSS 13.0, Chicago, IL, USA) was introduced for statistical analysis. Categorical data was examined by Pearson’s Chi-Square or Fisher’s exact test. Non-parametric Wilcoxon rank-sum test was adopted to analyze numerical variables. Linear regression as well as binary logistic regression were performed for multivariate analysis. The difference was statistically significant if two-sided *p* values < 0.05.

## Results

### Patient demographics and pathological evaluation

Based on our selection criteria, data for 1757 of 1948 patients (90.2%) were available: 1191 (67.8%) men and 566 (32.2%) females; median age: 62 (range, 22–90) years; 1121 (63.8%) distal, 609 total (34.7%) and 27 proximal (1.5%) gastrectomies. Neoadjuvant therapy was administered to 249 (14.2%) patients.

Microsatellite status was available for all 1757 specimens; MSI-H was found in 185 (10.5%) patients. MSI-H GC was found more frequently among female and elderly patients and more often in tumors located in the gastric antrum and pylorus (*p* < 0.001 for all). No statistically significant difference was found between the two groups with regard to the proportion of patients undergoing neoadjuvant therapy (*p* = 0.166). Statistically significant differences were found in several pathological characteristics of MSI-H, such as larger tumor size (*p* < 0.001), more well-differentiated adenocarcinoma (*p* < 0.001), lymphovascular emboli (*p* = 0.002), N_0_ stage (*p* < 0.001), NLNC (*p* < 0.001), and M_0_ stage (*p* = 0.002), and fewer perineural invasion (*p* < 0.001), tumor deposit (*p* = 0.009), and PLNC(*p* < 0.001) (Table 1).

**Table 1:** Patient demographics and pathological evaluation

	MSI-H GC	MSS GC	
	*n* = 185 (10.5%)	*n* = 1572 (89.5%)	*p* value
Sex, n (%)			*< 0.001*
Male	103 (55.7)	1088 (69.2)	
Female	82 (44.3)	484 (30.8)	
Age [y], median (quartile)	67 (60–73)	62 (54–69)	*< 0.001*
Tumor location, n (%)			*< 0.001*
Antrum and pylorus	125 (67.6)	713 (45.4)	
Angular	23 (12.4)	184 (11.7)	
Corpus	22 (11.9)	366 (23.3)	
Fundus and cardia	15 (8.1)	309 (19.7)	
Neoadjuvant therapy, n (%)			0.166
No	165 (89.2)	1343 (85.4)	
Yes	20 (10.8)	229 (14.6)	
Extent of lymphadenectomy			0.499
D1+ lymphadenectomy	22 (11.9)	221 (14.1)	
D2 lymphadenectomy	163 (88.1)	1351 (85.9)	
Tumor size [cm], median (quartile)	4 (2–6.5)	3 (2–4.8)	*< 0.001*
Pathology type, n (%)			*< 0.001*
Well-differentiated adenocarcinoma	66 (35.7)	425 (27.0)	
Moderately and poor-differentiated adenocarcinoma	67 (36.2)	554 (35.2)	
Mucinous adenocarcinoma	31 (16.8)	100 (6.4)	
Signet ring cell carcinoma	21 (11.4)	493 (31.4)	
Tumor stage, n (%)			0.205
Early GC	47 (25.4)	470 (29.9)	
Advanced GC	138 (74.6)	1102 (70.1)	
T stage, n (%)			0.600
T_is_ and T_1_	47 (25.4)	470 (29.9)	
T_2_	36 (19.5)	236 (15.0)	
T_3_	34 (18.4)	167 (10.6)	
T_4_	68 (36.8)	699 (44.5)	
Lymphovascular emboli, n (%)			*0.002*
No	97 (52.4)	1008 (64.1)	
Yes	88 (47.6)	564 (35.9)	
Perineural invasion, n (%)			*< 0.001*
No	138 (74.6)	955 (60.8)	
Yes	47 (25.4)	617 (39.2)	
Tumor deposit, n (%)			*0.009*
No	180 (97.3)	1445 (91.9)	
Yes	5 (2.7)	127 (8.1)	
PLNC, median (quartile)	0 (0–3)	2 (0–7)	*< 0.001*
NLNC, median (quartile)	27 (21–35)	23 (16–32)	*< 0.001*
N stage, n (%)			*< 0.001*
N_0_	95 (51.4)	630 (40.1)	
N_1_	33 (17.8)	239 (15.2)	
N_2_	30 (16.2)	276 (17.6)	
N_3a_	19 (10.3)	259 (16.5)	
N_3b_	8 (4.3)	168 (10.7)	
M stage, n (%)			*0.002*
M_0_	185 (100.0)	1495 (95.1)	
M_1_	0 (0.0)	77 (4.9)	
AJCC stage, n (%)			*0.005*
I	68 (36.8)	545 (38.9)	
II	57 (30.8)	298 (19.1)	
III	60 (32.4)	652 (40.4)	
IV	0 (0.0)	77 (4.9)	

*MSI* microsatellite instability

*MSS* microsatellite stability

*GC* gastric cancer

*PLNC* positive lymph node count

*NLNC* negative lymph node count

### LN count and adequate LN ratio

In the complete cohort of 1757 GC, a median of 28 LNs was retrieved, and the overall adequate LN ratio was 92.3% (1621/1757). Statistically significant differences were found in the median LN count (30 vs. 28, *p =* 0.031), but not in the adequate LN ratio (95.1% vs. 91.9%, *p =* 0.145) according to MSI-H and MSS, respectively. When restricted to distal gastrectomy (DG), a statistically significant difference was seen in the median LN count (30 vs. 27, *p =* 0.002), but not in the adequate LN ratio (95.6% vs. 91.2%, *p =* 0.061). When DGs were stratified according to AJCC stage, a statistically significant association between microsatellite status and median LN count was found only for AJCC stage II cancers (34 vs. 28, *p =* 0.005). Median LN counts did not differ statistically significantly between the two groups according to T category. A statistically significant difference in median LN count was found in patients undergoing DG for N_0_ cancers (28 vs. 24, *p =* 0.002). Conversely, no statistically significant difference was found in either the median LN count or the adequate LN ratio regardless of the AJCC Stage, T category and N category for patients undergoing total gastrectomy (TG) (Table 2). Further univariate and multivariate analysis demonstrated that MSI-H was an independent factor for total LN count in patients undergoing DG (B = 2.468, 95% CI 0.563 to 4.374, *p =* 0.011) (Table 3), and so it was in patients undergoing DG who were AJCC stage II (B = 5.105, 95% CI 1.432 to 8.779, *p =* 0.007) or N_0_ (B = 2.836, 95% CI 0.160 to 5.513, *p =* 0.038). MSI-H was not statistically correlated to the adequate LN ratio according to our univariate and multivariate analysis.

**Table 2:** Lymph node count and adequate lymph node ratio

	Lymph node harvest of MSI-H GC		Lymph node harvest of MSS GC		*p* value	
	Adequate ratio	Median (quartile)	Adequate ratio	Median (quartile)	Adequate ratio	Median (quartile)
All patients	95.1%	30(23–38)	91.9%	28(21–37)	0.145	*0.031*
Distal gastrectomy	95.6%	30(23–38)	91.2%	27(20–35)	0.061	*0.002*
Total gastrectomy	91.7%	30(25–39)	94.2%	30(22–41)	0.647	0.962
With NAdj therapy	95.0%	36(30–44)	93.4%	34(24–44)	1.000	0.395
W/out NAdj therapy	95.2%	29(23–38)	91.7%	27(20–36)	0.129	*0.034*
For Distal gastrectomy only						
AJCC Stage						
I	94.6%	27(21–33)	88.7%	24(19–33)	0.246	0.200
II	98.0%	34(26–42)	88.4%	28(19–36)	0.052	*0.005*
III	96.1%	31(25–38)	95.1%	29(23–37)	1.000	0.278
IV	N.A.	N.A.	94.1%	28(20–39)	N.A.	N.A.
T category						
T_is_ and T_1_	94.6%	25(19–33)	89.5%	26(19–30)	0.563	0.985
T_2_	97.1%	33(23–42)	89.2%	27(20–36)	0.206	0.056
T_3_	96.6%	35(26–41)	97.6%	30(24–37)	1.000	0.071
T_4_	94.9%	31(24–38)	92.5%	28(22–37)	0.783	0.086
N category						
N_0_	95.0%	28(22–36)	88.0%	24(19–33)	0.078	*0.002*
N_+_	96.2%	31(24–38)	93.8%	28(22–37)	0.607	0.083
For Total gastrectomy only						
AJCC Stage						
I	93.3%	26(17–30)	80.0%	26(20–34)	0.172	0.480
II	100.0%	37(27–46)	97.4%	30(21–41)	1.000	0365
III	100.0%	37(29–39)	94.8%	32(24–43)	0.364	0.618
IV	N.A.	N.A.	86.7%	29(19–43)	N.A.	N.A.
T category						
T_is_ and T_1_	75.0%	24(16–28)	94.3%	28(21–35)	0.104	0.122
T_2_	100.0%	26(26–40)	95.9%	27(20–35)	1.000	0.277
T_3_	100.0%	39(36–41)	96.1%	32(24–43)	1.000	0.273
T_4_	100.0%	30(27–42)	93.4%	32(22–43)	1.000	0.839
N category						
N_0_	92.3%	26(21–42)	93.4%	28(20–37)	0.602	0.738
N_+_	90.9%	32(29–39)	94.5%	32(23–43)	0.472	0.995

*MSI* microsatellite instability

*MSS* microsatellite stability

*GC* gastric cancer

*W/out* without

*NAdj* neoadjuvant

*N.A*. not applicable

**Table 3:** Univariate and multivariate analysis for lymph node count in patients undergoing distal gastrectomy

Factors	Univariate analysis		Multivariate analysis	
	B value	*p* value	B value (95% CI)	*p* value
Sex (M/F)	1.278	0.072	N.A.	N.A.
Age [y] (≤65/> 65)	− 1.225	0.080	N.A.	N.A.
Neoadjuvant therapy (N/Y)	4.444	*< 0.001*	3.562 (1.178,5.947)	*0.003*
Tumor size [cm] (≤3/> 3)	3.381	*< 0.001*	1.947 (0.353,3.540)	*0.017*
T stage (T_≤2_/T_3–4_)	2.863	*< 0.001*	−1.419 (−3.722,0.884)	0.227
Lymphovascular emboli (N/Y)	1.231	0.084	N.A.	N.A.
Perineural invasion (N/Y)	2.377	*0.001*	0.871 (−0.830,2.572)	0.316
Tumor deposit (N/Y)	1.959	0.182	N.A.	N.A.
pN stage (N_0_/N_+_)	3.474	*< 0.001*	1.528 (− 0.460,3.516)	0.132
M stage (M_0_/M_1_)	0.123	0.950	N.A.	N.A.
AJCC stage (I/II-IV)	3.854	*< 0.001*	2.073 (−0.755,4.900)	0.151
Extent of lymphadenectomy (D1+/D2)	3.248	*< 0.001*	0.037 (−1.995,2.068)	0.972
Microsatellite status (MSS/MSI-H)	2.720	*0.005*	2.468 (0.563,4.374)	*0.011*

*CI* confidence interval

*N.A*. not applicable

*MSI-H* microsatellite instability-high

*MSS* microsatellite stability

### LODDS and TLNT_(x%)_

A statistically significant difference in LODDS was found between MSI-H GC and MSS GC (Mean: − 5.017 vs. -2.727, *p <* 0.001, Wilcoxon rank-sum test). As shown in Table 4, to achieve TLNT_(90%)_ during pathological examination, 31 LNs are required to be tested for MSI-H GC, instead of 25 LNs for MSS GC. We found that TLNT_(X%)_ of MSI-H GC was always higher than that of MSS GC regardless of the given value of X%. If we set TLNT at 16 based on the current guideline for lymphadenectomy [4], LODDS would be − 2.619, implicating that 25.6% of LN_+_ MSI-H cases and 19.1% of LN_+_ MSS cases would be missed during pathological evaluation.

**Table 4:** LODDS and TLNT_(x%)_ at a given percentile of lymph node positive cases captured by pathological examination

Percentile of lymph node positive cases captured (x%)	MSI-H GC		MSS GC	
	LODDS	TLNT_(x%)_	LODDS	TLNT_(x%)_
99%	−3.819	51	−3.748	48
95%	−3.662	44	−3.430	35
90%	−3.309	31	−3.087	25
85%	−3.180	27	−2.801	19
80%	−2.953	22	−2.559	15
75%	−2.630	16	−2.354	12
50%	−1.977	9	−1.326	5

*MSI-H* microsatellite instability-high

*MSS* microsatellite stability

*GC* gastric cancer

*LODDS* log odds of positive lymph nodes to negative lymph nodes

*TLNT* target lymph node examined threshold

## Discussion

In this study, we found a statistically significant association between MSI-H and a higher LN count after curative gastrectomy, especially in patients undergoing DG and in patients undergoing DG who were AJCC stage II and N_0_. The absence of any statistically significant difference in LN count for patients undergoing TG could be partially explained by the low prevalence of MSI-H (24/609 = 3.9%) in this subgroup. On the other hand, the microsatellite status has no correlation with the adequate LN ratio irrespective of the extent of resection. However, TLNT_(X%)_ of MSI-H GC was always higher than that of MSS GC regardless of the given value of X%. We speculate that a greater lymph node yield is required during pathological examination in MSI-H GC in order to capture adequate LN_+_ cases.

High LN harvest after curative gastrectomy for GC has been found to be associated with better 5-year survival [11, 12]. Conversely, insufficient LN retrieval might lead to inaccurate staging [4, 13]. Several factors have been reported to impact LN yield during gastrectomy, such as age [14–16], ethnicity [14, 16], body mass index [17], tumor stage at diagnosis [14, 16, 18], institution volume [14, 15], and obviously, the extent of resection (TG vs. DG) [3, 19]. Of note, however, none of the above-mentioned publications analyzed genomic characteristics of GC. GCs can be classified into different molecular subtypes although the number of subtypes remains controversial and unclarified [9, 20]. GC with MSI-H phenotype is, without any doubt, one of the main subtypes due to its distinct histopathological patterns and particular clinical features [9, 21].

MSI-H prevalence in our study (10.5%) is in accordance with the literature for Asians (8.3%) [22], and close to the 9% overall rate reported in the meta-analysis by Polom et al. [23], but lower than those in worldwide genomic analysis studies (23 and 22%, respectively) [9, 24]. Indeed, MSI-H prevalence in Western patients varies from 8 to 45% [25, 26]. Several publications have underscored this difference between Asiatic patients and Western patients [21]. Apart from ethnicity, the lack of a standardized and quality-controlled diagnostic algorithm of MSI-H might be responsible for this variation in prevalence [25].

Several of the well-known associations between clinical/pathological features of GC and MSI-H were confirmed in our study, including elder age [22, 27–29], female gender [22, 27–29], occurrence in the distal stomach [22, 27, 28], larger tumor size [22], more mucinous pathology type [22], more lymphovascular emboli [22], limited LN involvement [22, 23, 25, 30], less advanced AJCC stage [25, 30], and in particular, a higher total LN count in MSI-H patients undergoing DG for AJCC stage II tumors [29].

However, conversely, Kim et al. [22] found a larger proportion of T_2–4_ tumors and more perineural invasion in MSI-H GC. Oki et al. [31] postulated that LN involvement was positively correlated to MSI-H GC. We speculate that the small sample size (414 and 240 cases, respectively) of these two studies and the high molecular heterogeneity of GC could partially explain these differences.

Our findings have several points in common with CRC studies: increased LN yield was strongly related to tumor location, tumor stage and microsatellite status [6, 7]. According to a location-specific analysis, no statistically significant difference in adequate LN was found between MSI-H and MSS tumors in the proximal colon [8]. In a further subclassification by UICC stage, a statistically significant association between MSI-H and higher LN count as well as higher adequate LN ratio could only be observed in stage I-II CRC (N_0_ tumors) but not in stage III CRC (N_+_ tumors) [8]. Moreover, lymphatic spread was less likely in MSI-H than MSS tumors [32]. Buckowitz et al. [33] found that microsatellite status was an independent predictor of distant metastases and attributed this finding to local lymphocyte infiltration in MSI-H CRCs.

The reasons for which MSI-H is associated with a higher total LN count in LN-negative stages in GC or CRC are difficult to ascertain for the moment. One reasonable explanation might be in the underlying molecular biological mechanism of MSI: the higher mutational rate in MSI-H tumors could potentially encode non-self immunogenic neoepitopes, which are known to induce an intense immune reaction and recruitment of lymphocytes [21]. This remarkably strong lymphocytic infiltration is described as “Crohn’s like” lymphoid reaction in CRC with MSI, a specific feature of this cancer type [8, 33]. As the size of LNs in MSI-H cancers was larger than that in MSS cancers [34], this could facilitate LN detection during the gross examination. However, in LN_+_ stages, this influence of tumor immunogenicity is overshadowed by a greater inflammatory response caused by tumor-induced tissue destruction and environmental invasion [8]. As neoadjuvant therapy may attenuate this MSI-related immune reaction, this might explain why no statistically significant difference was found between the MSI-H and MSS groups in the LN count in patients receiving neoadjuvant therapy.

Emerging data seem to indicate that MSI-H status has a favorable prognosis [21, 29, 30]. Polom et al. speculated that MSI-H tumors showed a high rate of N_0_ stage, a lower number of lymph node metastases, and a less extensive spread to lymph node stations than MSS tumors [35]. Since MSI-H GC has higher NLNC and lower PLNC, it is easy to know that more LNs need to be yielded by pathologist to avoid missing LN_+_ MSI-H GC, leading to a higher TLNT_(X%)_ compared to MSS GC. TLNT_(X%)_ was used to investigate the adequacy of pathological yield of LNs in previous studies [36, 37]. If the TLNT for MSI-H and MSS tumors were set at the same level, we can imagine that a larger proportion of LN_+_ cases would be missed in MSI-H GC compared to MSS GC. Thus, we speculate that a greater lymph node yield is required during pathological examination in MSI-H GC but this needs to be verified by further investigation.

Among the strengths of our study, this was, to the best of our knowledge, the first assessment of the potential association between MSI-H and LN yield in GC. Secondly, the study was conducted in a Chinese population, where the incidence of GC is relatively high. Thirdly, the sample size was large (1757 patients), increasing the feasibility of stratified and multivariate analysis to reduce the effect of confounding factors, such as the extent of resection.

Our study has several limitations. Firstly, this was a retrospective observational single-center study, only including Chinese patients. Secondly, MSI-H was detected by PCR method only when dMMR status was confirmed by IHC method. However, previous studies have shown a good correlation between the IHC and PCR method [10, 38]. Thirdly, microsatellite status of positive LNs was not tested, ignoring the probable heterogeneity between primary tumor and metastatic LNs [25, 39]. Lastly, LN counts depend on the type of surgical operation performed, the extended character of LN dissection of each individual surgeon, as well as the diligence with which pathologists search for LNs [3, 40].

## Conclusions

MSI-H was associated with higher LN yield in patients undergoing curative gastrectomy, especially for LN-negative GC. Although MSI-H did not affect the adequacy of LN harvest, a greater LN yield during pathological examination is suggested to capture adequate LN_+_ cases in MSI-H GC. Further investigations concerning the prognostic value of LN count in GC patients should be conducted once long-term survival information of these patients has been obtained.

## Data Availability

The datasets used and/or analysed during the current study are available from the corresponding author on reasonable request.

## Abbreviations

GC: Gastric cancer
LN: Lymph node
CRC: Colorectal cancer
MSI-H: Microsatellite instability-high
MMR: Mismatch repair
IHC: Immunohistochemical
PCR: Polymerase chain reaction
MSS: Microsatellite stability
LODDS: Log odds of positive lymph nodes to negative lymph nodes
PLNC: Positive lymph node count
NLNC: Negative lymph node count
TLNT: Target lymph node examined threshold
DG: Distal gastrectomy
TG: Total gastrectomy
CI: Confidence interval
